# Hypolipidemic Effects of *Alpinia japonica* Extracts: Modulation of PPAR Signaling, Gut Microbiota, and Intestinal Barrier Function in Hyperlipidemic Rats

**DOI:** 10.3390/ph18091320

**Published:** 2025-09-03

**Authors:** Liqing Zhou, Cong Fang, Hongwei Li, Yifan Lin, Huiqing Que, Hongxu Liu, Lihong Ma, Wenjin Lin

**Affiliations:** 1 Fujian Institute for Food and Drug Quality Control, Fuzhou 350012, China; fjifdczlq@163.com (L.Z.);; 2 College of Pharmacy, Fujian University of Traditional Chinese Medicine, Fuzhou 350122, China; fangcong0823@163.com (C.F.);; 3 Fujian Key Laboratory of Medical Analysis, Fujian Academy of Medical Sciences, Fuzhou 350001, Chinaliuhongxu@fjms.ac.cn (H.L.); lihongma@fjms.ac.cn (L.M.); 4 College of Bee Science and Biomedicine, Fujian Agriculture and Forestry University, Fuzhou 350002, China

**Keywords:** *Alpinia japonica*, hypolipidemic effects, transcriptomics, PPAR pathway, LC-MS, network pharmacology

## Abstract

**Objectives:** *Alpinia japonica* (*A. japonica*) is traditionally used for digestive disorders, but its hypolipidemic mechanisms remain unclear. This study investigated the lipid-lowering effects of its fruit (SJGS), rhizome (SJGJ), and leaf (SJY) extracts, exploring their bioactive constituents and organ-specific mechanisms. **Methods:** Sprague Dawley rats (n = 8/group) fed a high-fat diet received SJGS, SJGJ, or SJY (200 mg/kg/day) for 4 weeks. Serum lipids (TC, TG), liver enzymes (AST, ALT), and intestinal barrier markers (DAO) were measured. Gut microbiota (16S rDNA sequencing), hepatic histopathology, and ileal tight junction proteins were analyzed. Transcriptomics and qPCR assessed ileal gene expression. LC-MS identified chemical constituents, while network pharmacology predicted compound-target interactions. **Results:** All extracts significantly reduced serum TC (↓ 27–33%), TG (↓ 29–38%), AST/ALT (↓ 22–30%), and DAO (↓ 35–42%) versus controls (*p* < 0.05). They improved hepatic steatosis, enhanced intestinal barrier function, and modulated gut microbiota (↑ α-diversity, ↓ Firmicutes/Bacteroidetes ratio). Transcriptomics revealed PPAR signaling as the core pathway: SJGS/SJGJ downregulated fatty acid oxidation genes (*ACSL1*, *ACOX1*, *ACADM*), while SJY upregulated APOA1 (2.3-fold). LC-MS identified 33–48 compounds/part, with seven shared constituents. Network analysis prioritized three flavonoids (pinocembrin, luteolin, galangin) targeting TNF, AKT1, and PPAR pathways. **Conclusions:** The findings suggest *A. japonica* extracts ameliorate hyperlipidemia through distinct mechanisms—SJGS/SJGJ may inhibit fatty acid oxidation, while SJY potentially enhances APOA1-mediated clearance. Shared flavonoids likely contribute to these effects via PPAR signaling, supporting its traditional use. This study provides a scientific basis for the sustainable utilization of *A. japonica* resources.

## 1. Introduction

The rhizome of *Alpinia japonica* (Thunb.) Miq. (*A. japonica*), commonly known as Shānjiāng in traditional Chinese medicine, has been employed for centuries in East Asian ethnomedicine for managing gastrointestinal and inflammatory conditions [1]. *A. japonica* has been pharmacologically documented since the 6th century CE, when Tao Hongjing’s Collected Commentaries on the Shennong’s Classic of Materia Medica first recorded its dual therapeutic properties for cold-type abdominal pain and fasting support [2]. This early pharmacopeial description was later expanded in Li Shizhen’s Bencao Gangmu (Compendium of Materia Medica, Ming Dynasty, 16th century), which detailed its botanical characteristics—ginger-like leaves, pungent red flowers, and seeds resembling Alpinia katsumadai [2]. The Ming text further validated the plant’s traditional applications, particularly noting its processed formulations’ effectiveness against acute gastroenteritis and epigastric pain, while also documenting the flowers’ and seeds’ pungent-warm property and non-toxic characteristics. These recorded ethnopharmacological uses, preserved in classical Chinese medical literature, provide valuable insights into early applications of Alpinia species in gastrointestinal disorder management and metabolic modulation, warranting further phytochemical and pharmacological investigation.

Recent studies highlight *A. japonica* as a rich source of bioactive terpenoids, flavonoids, and essential oils with multifaceted pharmacological properties. Zhao et al. [3] demonstrated that its rhizome volatile oil enhances gastrointestinal motility in mice. GC-MS analyses identified key monoterpenes, including (1*S*,4*R*)-(+)-camphor and 1,8-cineole, as dominant constituents [4]. Anticancer research revealed that its aqueous extract induces G0/G1 arrest and JNK-mediated apoptosis in HepG2 cells [5]. Anti-inflammatory sesquiterpenoids (e.g., xanthorrhizol) from rhizomes suppress NO production in macrophages [6,7], while seed-derived alpinone 3-acetate modulates 155 genes, showing efficacy comparable to hydrocortisone in edema models [8]. Notably, the characteristic compound 4-hydroxyboesenbergin B disrupts vancomycin-resistant Enterococcus biofilms and restores gut microbiota balance by promoting Lactobacillus [9]. These findings underscore *A. japonica*’s potential for treating metabolic, inflammatory, and infectious diseases, providing a mechanistic basis for its clinical development.

Hyperlipidemia, a key risk factor for cardiovascular diseases, involves complex pathophysiology, including hepatic lipid dysregulation, gut microbiota imbalance, and intestinal barrier dysfunction [10,11]. Current synthetic lipid-lowering drugs (e.g., statins) often cause adverse effects like myalgias and an increased risk of new-onset diabetes [11,12], driving the search for safer alternatives to traditional medicines. Traditional Chinese Medicine (TCM), capable of simultaneously modulating gut microbiota and lipid metabolism, has gained attention [13,14]. Systematic reviews highlight that probiotics and TCM targeting the gut–liver axis demonstrate superior efficacy over monotherapies, with Zingiberaceae plants like turmeric showing particular promise due to their unique bioactive properties [10]. Notably, other Alpinia species have demonstrated anti-obesity, hypolipidemic, hypoglycemic, and hepatoprotective effects activity [15,16,17,18], hinting at a possible conserved mechanism within the genus. Nevertheless, no studies have systematically compared the chemical profiles of *A. japonica* medicinal parts or linked specific constituents to hypolipidemic targets.

Here, we bridge ethnopharmacological wisdom with multi-omics strategies to systematically investigate *A. japonica*’s lipid-lowering potential. Through a combination of in vivo validation, transcriptomic profiling, and gut microbiota analysis, we demonstrate for the first time that fruit, rhizome, and leaf extracts exert complementary hypolipidemic effects in a high-fat diet model. LC-MS-based metabolomics coupled with network pharmacology reveals that, while all three parts share core flavonoid agonists targeting PPAR signaling, they differentially regulate key metabolic nodes. These findings not only substantiate *A. japonica*’s traditional use in lipid disorders but also provide a molecular framework for rationalizing its whole-plant utilization in metabolic syndrome management.

## 2. Results

### 2.1. Effects of A. japonica on Metabolic Parameters in High-Fat Diet-Fed Rats

**Figure 1:**
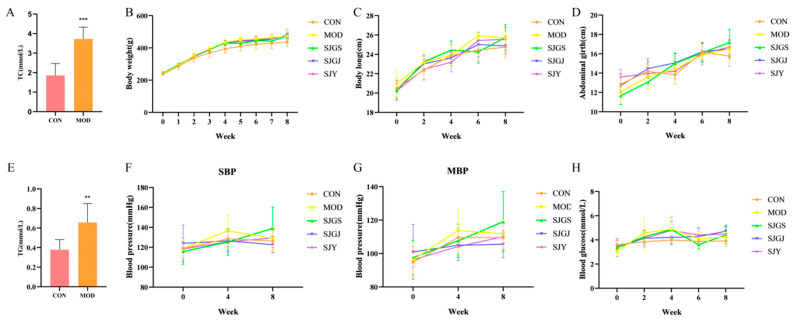
Effects of different parts of *A. japonica* on body weight, body length, abdominal girth, blood pressure, and blood glucose in high-fat diet-fed rats (n = 8). (**A**) Changes in serum total cholesterol (TC) levels in rats at week 4; (**B**) Changes in body weight; (**C**) Changes in body length; (**D**) Changes in abdominal girth; (**E**) Changes in serum total cholesterol triglyceride (TG) levels in rats at week 4; (**F**) Changes in systolic blood pressure (SBP); (**G**) Changes in mean blood pressure (MBP). (**H**) Changes in blood glucose. CON, Normal control group; MOD, Model group; SJGS, *A. japonica* fruit group; SJGJ, *A. japonica* rhizome group; SJY, *A. japonica* leaf group; ** *p* < 0.01, *** *p* < 0.001 vs. MOD.

The hyperlipidemic rat model was successfully induced, as evidenced by significantly elevated serum TC and TG levels in MOD compared to CON (*p* < 0.001) after 4 weeks of high-fat diet feeding (Figure 1A,E). MOD rats exhibited characteristic metabolic disturbances, including lethargy, reduced activity, dull fur, and increased food/water intake with polyuria and oily stools, while these symptoms were ameliorated in treatment groups (SJGS, SJGJ, SJY).

Quantitative analysis revealed significantly lower food intake in the MOD (20.29 g/day) and treatment groups (20.31, 19.91, 19.79 g/day) compared with CON (26.59 g/day, *p* < 0.05), though no significant differences were observed among MOD and treatment groups (*p* > 0.05). Morphometric parameters (body weight, length, and abdominal girth) were comparable across groups at baseline (*p* > 0.05, Figure 1B–D). While all high-fat diet groups showed progressive increases in body weight, length, and abdominal girth during modeling, the treatments did not significantly affect these parameters compared to MOD (*p* > 0.05). Notably, neither the modeling protocol nor treatments significantly altered fasting blood glucose or blood pressure parameters (SBP, MBP) relative to CON (Figure 1F–H), confirming the specific induction of dyslipidemia without gluco-metabolic or cardiovascular perturbations.

### 2.2. Hepatoprotective and Lipid-Lowering Effects of A. japonica in High-Fat Diet-Fed Rats

**Figure 2:**
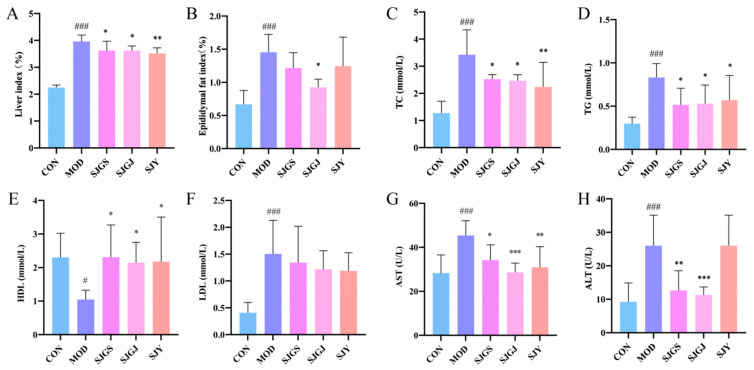
Effects of different parts of *A. japonica* on liver index, epididymal fat, TC, TG, HDL, LDL, AST, and ALT in high-fat diet-fed rats (n = 8). (**A**) Changes in liver index; (**B**) Changes in epididymal fat index; (**C**) Changes in TC levels; (**D**) Changes in TG levels; (**E**) Changes in HDL levels; (**F**) Changes in LDL levels; (**G**) Changes in AST levels; (**H**) Changes in ALT levels. CON, Normal control group; MOD, Model group; SJGS, *A. japonica* fruit group; SJGJ, *A. japonica* rhizome group; SJY, *A. japonica* leaf group; TC, Total cholesterol; TG, Triglyceride; HDL, High-density lipoprotein-C; LDL, Low-density lipoprotein-C; AST, Aspartate aminotransferase; ALT, Alanine aminotransferase; ^#^ *p* < 0.05, ^###^ *p* < 0.001 vs. CON; * *p* < 0.05, ** *p* < 0.01, *** *p* < 0.001 vs. MOD.

The high-fat diet significantly increased both liver index and epididymal fat index in the MOD group compared to CON (*p* < 0.001, Figure 2A,B), while all three treatments (SJGS, SJGJ, SJY) effectively reduced the liver index (*p* < 0.05, Figure 2A). Notably, SJGJ treatment significantly decreased epididymal fat index (*p* < 0.05, Figure 2B), with SJGS and SJY showing similar but non-significant trends.

Lipid profile analysis revealed the high-fat diet successfully induced hyperlipidemia, evidenced by higher levels of LDL-C, TC, and TG, but lower HDL-C in MOD versus CON (*p* < 0.05, Figure 2C–F). Compared with the model (MOD) group, high-fat diet-fed rats treated with SJGS, SJGJ, and SJY exhibited significantly reduced serum total cholesterol (TC) and triglyceride (TG) levels, along with increased high-density lipoprotein cholesterol (HDL) levels (*p* < 0.05). Notably, SJY demonstrated a superior TG-lowering effect compared to the other two herbal treatments (*p* < 0.01, Figure 2C–F). However, no statistically significant differences were observed in low-density lipoprotein cholesterol (LDL) levels among the SJGS, SJY, and SJGJ groups compared to the MOD group (*p* > 0.05).

Compared with CON, the MOD showed significantly elevated serum ALT and AST levels (*p* < 0.001, Figure 2G,H), indicating aggravated hepatocellular damage. After intervention with SJGS, SJGJ, and SJY, the serum AST levels in rats were reduced to varying degrees (*p* < 0.05, *p* < 0.001, and *p* < 0.01, respectively, Figure 2G,H). Moreover, both SJGS and SJGJ groups exhibited significant decreases in serum ALT levels (*p* < 0.01 and *p* < 0.001, respectively, Figure 2G,H). These findings suggest that the three parts of *A. japonica* may exert hepatoprotective effects by alleviating hepatocellular injury.

### 2.3. Histopathological Improvements of Hepatic Steatosis by A. japonica in High-Fat Diet-Fed Rats

**Figure 3:**
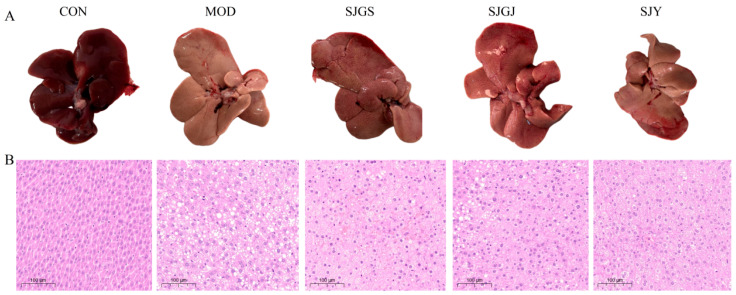
Effects of different parts of *A. japonica* on macroscopic appearance and H&E staining of liver tissues in high-fat diet-fed rats (n = 3). CON, Normal control group; MOD, Model group; SJGS, *A. japonica* fruit group; SJGJ, *A. japonica* rhizome group; SJY, *A. japonica* leaf group; (**A**) macroscopic appearance; (**B**) H&E staining of liver tissues.

Macroscopic examination revealed characteristic fatty liver changes in the MOD group, including yellowish discoloration, rough surface, rounded edges, and significant enlargement with tense capsule and greasy cut surface, while CON group livers maintained normal reddish coloration, smooth texture, and sharp margins. All three treatments (SJGS, SJGJ, and SJY) demonstrated dose-dependent improvements in liver color, size, and morphology (Figure 3A). Histopathological analysis confirmed these observations, with CON group hepatocytes showing normal architecture, abundant cytoplasm, and rare lipid vacuoles, whereas MOD group exhibited severe diffuse steatosis featuring numerous lipid vacuoles and displaced nuclei. Treatment groups showed marked reduction in lipid accumulation and architectural improvement, though not fully restoring normal histology (Figure 3B).

### 2.4. Effects of A. japonica on Intestinal Motility and Barrier Function in High-Fat Diet-Fed Rats

**Figure 4:**
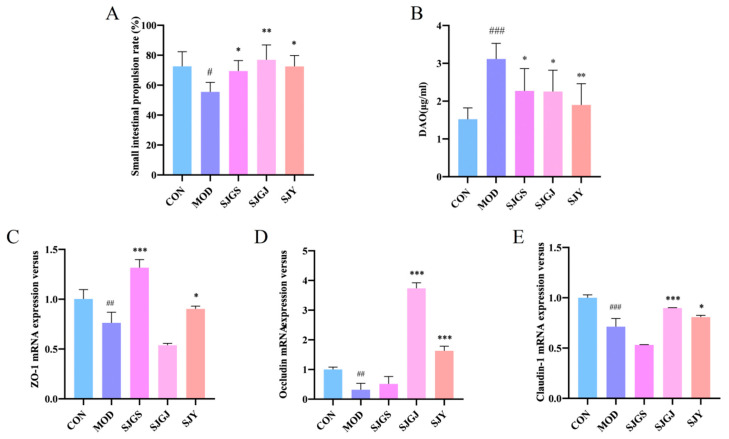
Effects of different parts of *A. japonica* on intestinal motility, DAO, *ZO-1*, *OCCLUDIN*, and *CLAUDIN* mRNA expression in high-fat diet-fed rats (n = 3). CON, Normal control group; MOD, Model group; SJGS, *A. japonica* fruit group; SJGJ, *A. japonica* rhizome group; SJY, *A. japonica* leaf group; (**A**) Changes in small intestinal propulsion rate; (**B**) Changes in Serum diamine oxidase (DAO) levels; (**C**) *ZO-1* mRNA expression; (**D**) *OCCLUDIN* mRNA expression; (**E**) *CLAUDIN* mRNA expression; ^#^ *p* < 0.05, ^##^ *p* < 0.01, ^###^ *p* < 0.001 vs. CON; * *p* < 0.05, ** *p* < 0.01, *** *p* < 0.001 vs. MOD.

The intestinal motility assessment revealed significantly reduced small intestinal propulsion rate in the MOD group compared to CON (*p* < 0.05, Figure 4A). Treatment with SJGJ not only restored but exceeded normal propulsion rates (*p* < 0.01 vs. MOD and CON, Figure 4A), while SJGS and SJY treatments showed intermediate improvement (higher than MOD but lower than CON, *p* < 0.05, Figure 4A). Serum diamine oxidase (DAO) levels, a sensitive biomarker of intestinal barrier integrity, were markedly elevated in the MOD group (*p* < 0.001 vs. CON, Figure 4B), indicating impaired intestinal permeability. All three treatments (SJGS, SJY, and SJGJ) significantly attenuated this increase (*p* < 0.05, *p* < 0.05, and *p* < 0.01, respectively, vs. MOD, Figure 4B), demonstrating their protective effects against hyperlipidemia-induced intestinal mucosal damage and permeability dysfunction.

### 2.5. Modulation of Intestinal Tight Junction Protein Expression by A. japonica in High-Fat Diet-Fed Rats

The qPCR analysis revealed significant downregulation of key tight junction proteins (*ZO-1*, *OCCLUDIN*, and *CLAUDIN*) in the ileal mucosa of the MOD group compared to CON (*p* < 0.05, Figure 4C–E), indicating impaired intestinal barrier integrity. Treatment with SJGS and SJY significantly upregulated *ZO-1* mRNA expression versus MOD (*p* < 0.05, Figure 4C), while SJGJ and SJY administration markedly increased both *CLAUDIN* and *OCCLUDIN* transcript levels (*p* < 0.05, Figure 4D,E). These findings demonstrate that all three treatments can enhance intestinal barrier function, potentially through differential upregulation of specific tight junction components—with SJY showing the broadest spectrum of activity across all three proteins analyzed. The coordinated increase in these structural proteins (*CLAUDIN*, *ZO-1*, and *OCCLUDIN*) provides a molecular mechanism for the observed reduction in intestinal permeability following treatment administration.

### 2.6. Modulation of Gut Microbiota Composition and Diversity by A. japonica in High-Fat Diet-Fed Rats

**Figure 5:**
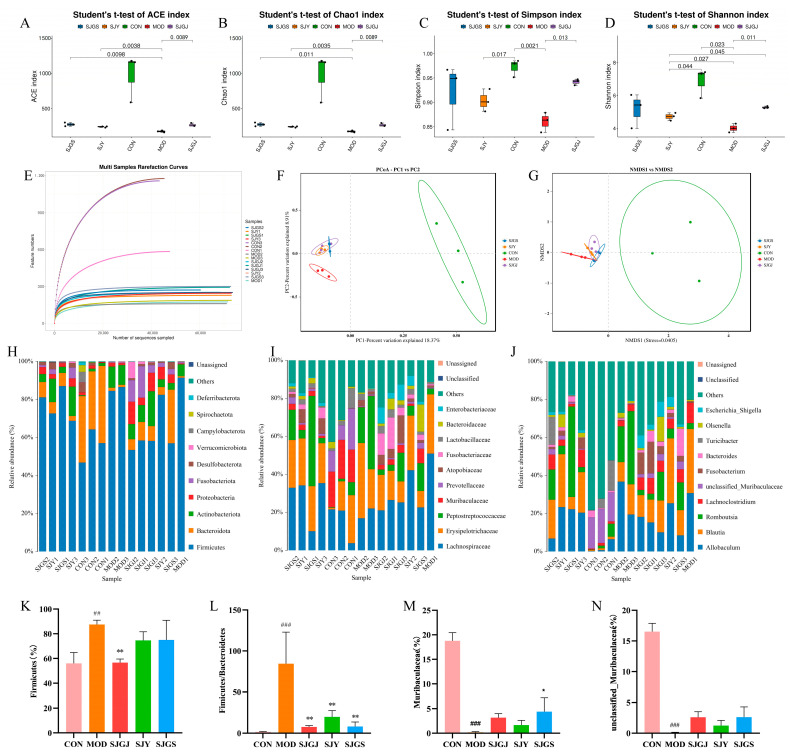
Effects of different parts of *A. japonica* on gut microbiota α-diversity and taxonomic composition at phylum, family, and genus levels in high-fat diet-fed rats (n = 3). (**A**) Gut microbiota ACE index; (**B**) Gut microbiota Chao1 index; (**C**) Gut microbiota Simpson index; (**D**) Gut microbiota Shannon index; (**E**) Sample rarefaction curves; (**F**) Principal Coordinates Analysis (PCoA) plot, the ellipses denote 95% confidence intervals; (**G**) Non-metric Multidimensional Scaling (NMDS) Plot, the ellipses denote 95% confidence intervals; (**H**) Microbial community composition at the phylum level; (**I**) Microbial community composition at the family level; (**J**) Microbial community composition at the genus level; (**K**) Firmicutes in rat gut microbiota; (**L**) Ratio of Firmicutes to Bacteroidetes; (**M**) Muribaculaceae abundance in gut microbiota; (**N**) Unclassified Muribaculaceae genus abundance in gut microbiota; CON, Normal control group; MOD, Model group; SJGS, *A. japonica* fruit group; SJGJ, *A. japonica* rhizome group; SJY, *A. japonica* leaf group; ^##^ *p* < 0.01, ^###^ *p* < 0.001 vs. CON; * *p* < 0.05, ** *p* < 0.01 vs. MOD.

Sequencing of 15 samples produced 1,093,874 quality-filtered reads after processing 1,186,477 raw paired-end reads (92.2% efficiency). Per-sample read counts ranged from 61,448 to 84,502 (mean 72,925), demonstrating sufficient coverage for downstream analyses (Supplementary Table S1). ASVs (Amplicon Sequence Variants) were obtained by denoising the sequences with the DADA2 method implemented in QIIME2 software (Supplementary Table S2). The gut microbiota analysis revealed significant alterations in microbial diversity and composition associated with hyperlipidemia. Alpha diversity indices (Chao1, Ace, Simpson, and Shannon) were markedly reduced in the MOD group compared to CON (*p* < 0.05, Figure 5A–D), indicating diet-induced microbial depletion. All three *A. japonica* extracts (SJGS, SJGJ, and SJY) effectively restored microbial richness, with SJY showing the most pronounced effects (*p* < 0.05, Figure 5A–D). The rarefaction curves demonstrated adequate sequencing saturation across all samples, confirming sufficient sequencing depth for robust microbial diversity analysis (Figure 5E).

The PCoA results demonstrated clear separation between the control group (CON) and the three *A. japonica* extract treatment groups (PC1 = 18.37%, PC2 = 8.91%), with the extract-treated groups clustering closely together (Figure 5F). The NMDS analysis (Stress = 0.0405) confirmed significant separation between the control (CON) and all *A. japonica* treatment groups, with tight within-group clustering indicating treatment-specific microbiome modulation (Figure 5G). At the phylum level, the MOD group exhibited characteristic dysbiosis patterns, including increased Firmicutes/Bacteroidota (F/B) ratio (*p* < 0.001, Figure 5L) and reduced Actinobacteriota abundance. Treatment groups consistently reversed these changes, significantly lowering the F/B ratio (*p* < 0.01, Figure 5H,K,L) and showing trends toward Bacteroidota enrichment. Finer taxonomic analysis demonstrated extract-mediated restoration of beneficial taxa, including the Muribaculaceae family (notably unclassified Muribaculaceae genus) that were depleted in the MOD group (*p* < 0.001, Figure 5I,J,M,N).

### 2.7. Transcriptomic Profiling Reveals DEGs Regulation by A. japonica in High-Fat Diet-Fed Rat Ileum

**Figure 6:**
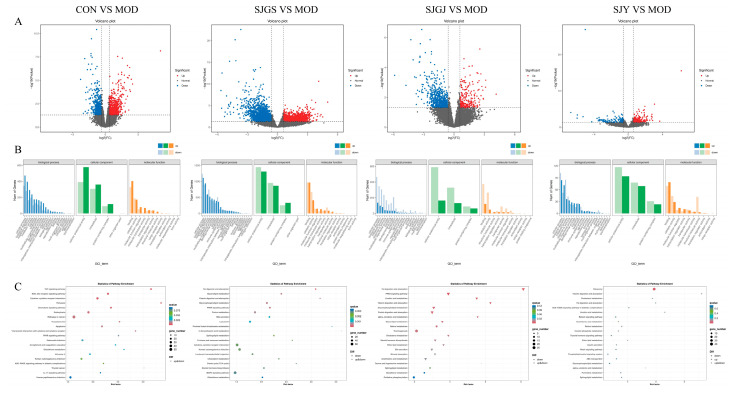
Effects of *A. japonica* on the ileal transcriptome in high-fat diet-fed rats. (**A**) Volcano plot; (**B**) GO enrichment; (**C**) KEGG pathway analysis. CON, Normal control group; MOD, Model group; SJGS, *A. japonica* fruit group; SJGJ, *A. japonica* rhizome group; SJY, *A. japonica* leaf group.

Comparative transcriptomic analysis among five groups (CON, MOD, SJGJ, SJY, and SJGS) was performed using pairwise comparisons, identifying distinct gene expression patterns with a threshold of |FC| ≥ 1.5 and FDR < 0.05 (Figure 6A). The CON vs. MOD comparison revealed 1310 differentially expressed genes (DEGs; 719 up-regulated, 591 down-regulated, Table 1), demonstrating extensive transcriptional remodeling in hyperlipidemia. Treatment-specific responses were observed: SJY vs. MOD showed 319 DEGs (176↑, 143↓, Table 1), SJGJ vs. MOD exhibited 976 DEGs (237↑, 739↓, Table 1), while SJGS vs. MOD displayed the most profound modulation with 3587 DEGs (1692↑, 1895↓, Table 1). GO enrichment analysis categorized these DEGs into key biological processes, including cellular metabolic processes (particularly lipid and energy metabolism), response to stimuli, and biological regulation, with molecular functions predominantly involving catalytic activity, binding, and molecular transducer activity (Figure 6B). KEGG pathway analysis revealed treatment-specific metabolic regulation: SJGJ significantly enriched cholesterol metabolism, PPAR signaling, and lipid digestion/absorption pathways; SJY preferentially modulated fat digestion and sphingolipid metabolism; while SJGS demonstrated the broadest spectrum of activity across PPAR signaling, ether lipid metabolism, and arachidonic acid pathways (Figure 6C). Notably, all treatments converged on core lipid metabolic pathways but with distinct transcriptional fingerprints, suggesting complementary mechanisms of action for different plant parts.

### 2.8. Validation of Lipid Metabolism-Related Gene Expression by A. japonica Extracts in High-Fat Diet-Fed Rats

**Figure 7:**
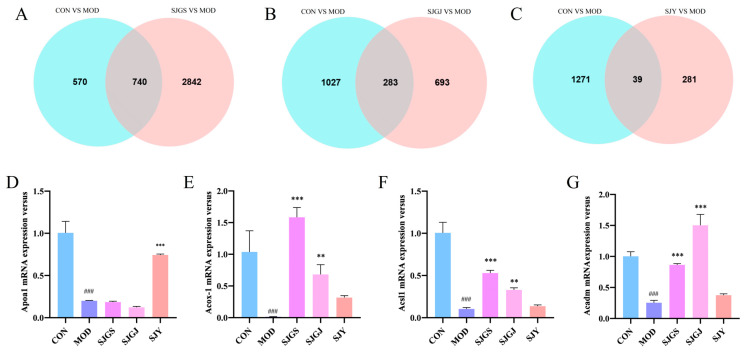
Analysis of DEGs regulation by *A. japonica* in the high-fat diet-fed rats’ ileum(n = 3). (**A**) Venn diagram of DEGs between CON vs. MOD and MOD vs. SJGS; (**B**) Venn diagram of DEGs between CON vs. MOD and MOD vs. SJGJ; (**C**) Venn diagram of DEGs between CON vs. MOD and MOD vs. SJY; (**D**) Relative expression of *APOA1* genes; (**E**) Relative expression of *ACSL1* genes; (**F**) Relative expression of *ACOX1* genes; (**G**) Relative expression of *ACADM* genes; Note: ^###^ *p* < 0.001 vs. normal group; ** *p* < 0.01 vs. model group; *** *p* < 0.001 vs. model group.

Transcriptomic intersection analysis identified 740, 283, and 39 overlapping differentially expressed genes (DEGs) between CON vs. MOD and MOD vs. SJGS, MOD vs. SJGJ, and MOD vs. SJY comparisons, respectively (Figure 7A–C), with four key lipid metabolism-related genes (*APOA1*, *ACSL1*, *ACOX1*, and *ACADM*) selected for qPCR validation. The qPCR results confirmed significant downregulation of all four genes in MOD versus CON (*p* < 0.05). Treatment with SJGS and SJGJ significantly upregulated *ACSL1*, *ACOX1*, and *ACADM* expression compared to MOD (*p* < 0.01, *p* < 0.001, respectively, Figure 7E–G), while SJY specifically enhanced APOA1 transcription (*p* < 0.001, Figure 7D) without affecting the other three genes. These findings demonstrate distinct regulatory patterns among the different *A. japonica* extracts, with SJGS and SJGJ primarily modulating fatty acid activation and oxidation genes *(ACSL1*, *ACOX1*, *ACADM*), whereas SJY selectively influenced apolipoprotein metabolism through APOA1 upregulation, providing mechanistic insights into their shared hypolipidemic effects through complementary molecular pathways.

### 2.9. Chemical Profiling Reveals Differential Phytochemical Composition Among A. japonica Plant Parts

**Figure 8:**
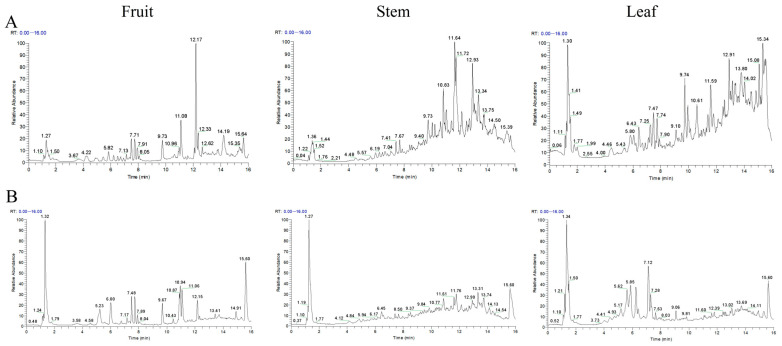
LC-MS total ion current chromatograms of phytochemicals in *A. japonica* fruit, rhizome, and leaves: (**A**) positive ion mode; (**B**) negative ion mode.

**Figure 9:**
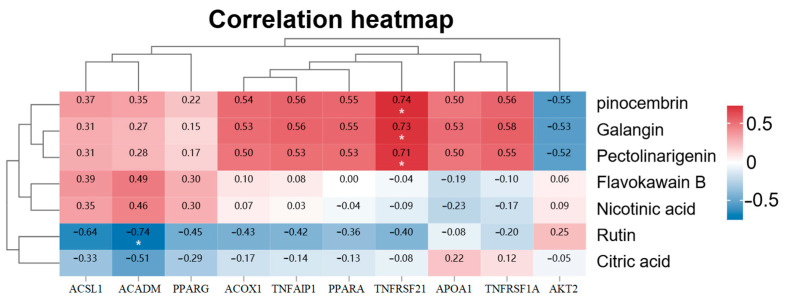
Pearson correlation analysis between common flavonoid compounds in *A. japonica* and PPAR-related genes. The asterisk (*) indicates a statistically significant correlation (*p* < 0.05).

LC-MS analysis in both positive and negative ion modes identified distinct phytochemical profiles across different plant parts of *A. japonica* (Figure 8A,B). The fruits contained 46 characterized compounds, including 23 flavonoids (e.g., formononetin, naringenin chalcone, and pinocembrin), 5 phenolic acids, 4 alkaloids, 3 terpenoids, and 2 fatty acids. The rhizomes yielded 33 compounds dominated by 13 flavonoids (particularly alpinetin, cardamonin, and pinocembrin) and 6 terpenoids (including parthenolide). Leaves showed the highest diversity with 48 compounds, featuring 22 flavonoids (notably quercetin-3-glucuronide) and 9 terpenoids. Identification and confidence level of chemical constituents in *A. japonica* extracts by UPLC-Q-Exactive Orbitrap-MS were provided in Supplementary Table S3. Seven common constituents were detected across all parts: pinocembrin, galangin, flavokawain B, nicotinic acid, pectolinarigenin, rutin, and citric acid. LC-MS total ion current chromatograms analysis (Figure 8) confirmed significant inter-part compositional differences, with fruits accumulating formononetin (7.2% relative abundance) and naringenin chalcone (5.8%), rhizomes rich in flavokawain B (6.5%) and alpinetin (5.3%), and leaves containing ferulic acid (8.1%) and quercetin-3-glucuronide (4.9%) as major components. Pearson correlation analysis between the seven shared components and ten key PPAR signaling pathway genes from transcriptome sequencing revealed that pinocembrin, galangin, and pectolinarigenin showed positive correlations with TNFRSF21 gene expression levels (r = 0.74, 0.73, and 0.71, respectively; all *p* < 0.05, Figure 9), while rutin exhibited a negative correlation with *ACADM* gene expression (r = −0.74, *p* < 0.05, Figure 9). These findings establish a chemical basis for observed differential pharmacological activities among plant parts.

### 2.10. Network Pharmacological Analysis Reveals Multi-Component Synergistic Mechanisms of A. japonica Against Hyperlipidemia

**Figure 10:**
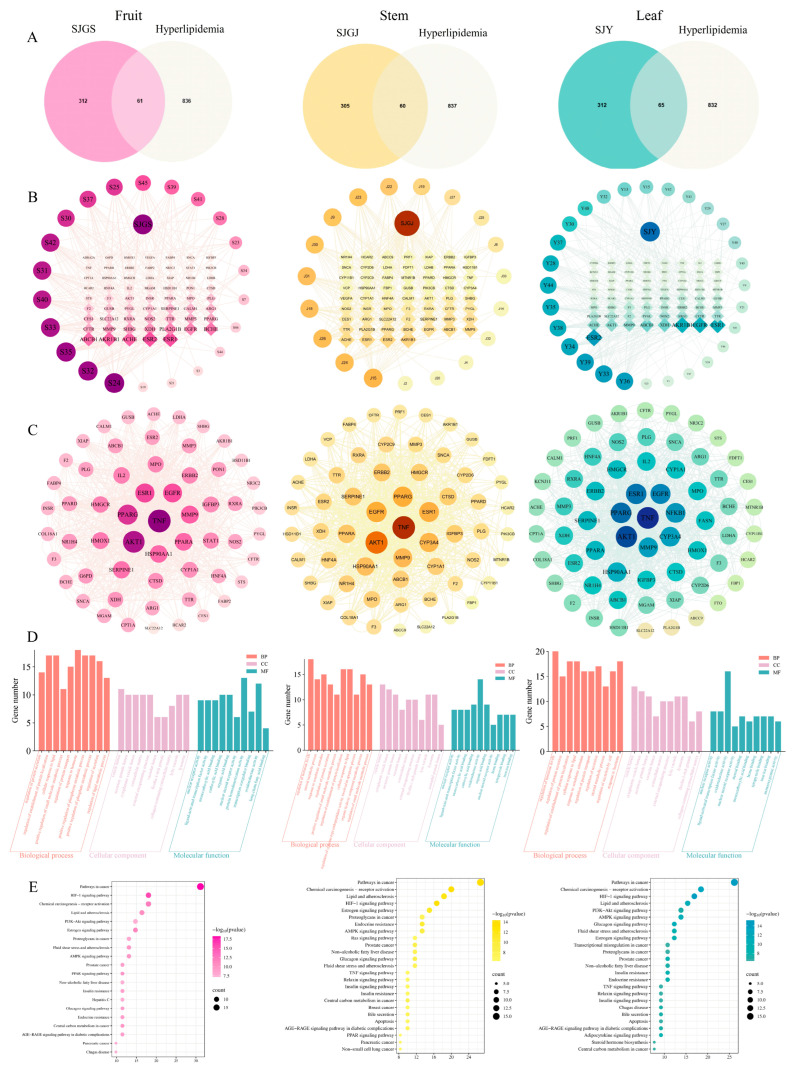
Multi-omics network analysis of *A. japonica* against hyperlipidemia. (**A**) Shared targets between different *A. japonica* parts and hyperlipidemia; (**B**) Network of “active components—shared targets” for different *A. japonica* parts; (**C**) Protein–protein interaction (PPI) network of *A. japonica*-related targets; (**D**) Gene Ontology enrichment analysis; (**E**) KEGG pathway enrichment analysis.

Comprehensive network pharmacology analysis identified 37, 28, and 36 bioactive components in fruits, rhizomes, and leaves of *A. japonica, respectively,* through SwissADME screening, with 373, 365, and 377 corresponding targets predicted by SwissTargetPrediction. Intersection with 897 hyperlipidemia-related targets from GeneCards yielded 61, 60, and 65 potential therapeutic targets for each plant part (Figure 10A). Compound-target networks constructed using Cytoscape 3.9.1 revealed distinct key components: fruits were characterized by naringenin chalcone and 7,8-dihydroxyflavone, rhizomes by naringenin and pectolinarigenin, and leaves by farrerol and naringenin chalcone(Figure 10B). Protein–protein interaction analysis identified TNF, AKT1, PPARG, and ESR1 as core targets across all preparations(Figure 10C). GO enrichment analysis (*p* < 0.01) demonstrated consistent involvement in hormonal response (BP), vesicle lumen (CC), and oxidoreductase activity (MF) for all extracts (Figure 10D). KEGG pathway enrichment analysis via Metascape (*p* < 0.01) revealed that SJGS exerted hypolipidemic effects by modulating PPAR, AMPK, PI3K-Akt, lipid metabolism, and non-alcoholic fatty liver disease pathways. SJGJ was primarily involved in PPAR, AMPK, and NAFLD pathways, while SJY significantly enriched AMPK, PI3K-Akt, and lipid metabolism pathways (Figure 10E).

## 3. Discussion

Our study suggests that *A. japonica* extracts may exert multifaceted therapeutic effects against high-fat diet-induced hyperlipidemia through integrated modulation of lipid metabolism, hepatic function, intestinal barrier integrity, and gut microbiota composition. These findings align with recent advances in metabolic disease research, demonstrating the complex interplay between dietary factors, gut microbiota, and host metabolism [19,20,21]. The successful induction of hyperlipidemia in our model system recapitulates key features of human metabolic syndrome, including dyslipidemia, hepatic steatosis, and gut dysbiosis, as described in foundational studies of diet-induced obesity [22,23,24]. Notably, the differential effects observed among plant parts (fruits, rhizomes, and leaves) provide novel insights into the phytochemical basis of traditional medicinal applications, building upon previous phytochemical characterizations of Zingiberaceae species [25]. These results may contribute to our understanding of botanical interventions for metabolic disorders by incorporating multi-omics approaches that bridge traditional knowledge with contemporary systems biology [26,27,28].

The lipid-modulating properties of *A. japonica* extracts appear to show organ-specific patterns, with leaf-derived SJY showing superior triglyceride-lowering effects, while rhizome and fruit extracts preferentially enhanced fatty acid oxidation pathways. This observed functional variation may correlate with distinct phytochemical profiles revealed by our LC-MS analysis and mirrors findings from other medicinal plants, where different organs contain unique bioactive compounds [29,30,31,32,33]. The upregulation of APOA1 by SJY could suggest potential enhancement of reverse cholesterol transport, a mechanism previously implicated in the cardioprotective effects of flavonoids [34]. Meanwhile, the coordinated induction of *ACSL1*, *ACOX1*, and *ACADM* by SJGJ and SJGS suggests enhanced mitochondrial β-oxidation, which aligns with prior reports on PPARα-mediated lipid metabolism regulation [35,36,37].

The hepatoprotective effects observed in this study assume particular significance in light of the growing NAFLD pandemic [38,39,40]. Our histological and biochemical findings demonstrate that *A. japonica* extracts appear to influence multiple aspects of diet-induced liver injury, including steatosis, inflammation, and hepatocellular damage. This comprehensive hepatoprotection resembles that reported for other PPARα-modulating botanicals [41,42], while also potentially affecting gut microbiota composition. The reduction in ALT/AST levels was particularly striking, exceeding effects seen with some clinically used hepatoprotectants in comparable models [43]. While complete histological normalization was not achieved, the degree of improvement suggests clinically relevant mitigation of liver damage, especially considering the relatively short treatment duration compared to natural history studies of NAFLD progression [44,45,46].

Our investigation of gut–liver axis modulation yields several novel insights with translational potential. The restoration of intestinal barrier function, evidenced by both biochemical (DAO) and molecular (tight junction proteins) markers, addresses a key pathological feature of metabolic disease that has recently gained recognition [47,48]. The differential regulation of specific junctional proteins by various extracts suggests complex, phytochemical-dependent mechanisms of barrier enhancement. Particularly noteworthy is SJGJ’s prokinetic effect, which may involve modulation of serotonin signaling or other neuroendocrine pathways known to influence gut motility [49,50,51]. These findings expand upon previous reports of gut barrier-protective botanicals by demonstrating organ-specific effects within a single plant species [52,53]. The microbiota analysis reveals another layer of complexity, suggesting that *A. japonica* may selectively enrich beneficial taxa like Muribaculaceae while normalizing the Firmicutes/Bacteroidetes ratio—a microbial signature associated with metabolic health [54,55]. This prebiotic-like activity may explain part of the extract’s systemic benefits through microbial metabolite signaling [56,57].

This study suggests that the hypolipidemic effects of *A. japonica* may be predominantly attributed to its flavonoid components acting through a multi-target network. The key bioactive constituents—pinocembrin (modulating PPARα pathway) [58], galangin (exhibiting anti-obesity effects) [59], and alpinetin (ameliorating oxidative stress) [60]—could collectively influence lipid metabolism via PPAR signaling and gut microbiota modulation. Network pharmacology analysis pinpointed TNF and AKT1 as potential pivotal targets, where TNF might mediate lipolysis through IL-6 regulation [61], while AKT1 may contribute to metabolic balance [62]. Notably, PPARγ emerged as a possible central regulator, with its activity potentially inhibited by specific plant components [63]. Intriguingly, organ-specific variations were observed: fruit and rhizome extracts activated PPAR pathways, whereas leaves exhibited distinct mechanisms, indicating differential bioactive distribution. These findings underscore *A. japonica*’s potential comprehensive therapeutic strategy against hyperlipidemia, integrating metabolic, anti-inflammatory, and microbiota-modulating effects. While our transcriptomic and qPCR data highlight distinct gene expression patterns associated with each extract, future studies employing targeted phytochemical isolation, reporter gene assays, or gene knockdown experiments are needed to establish direct mechanistic causality.

## 4. Materials and Methods

### 4.1. Chemicals and Reagents

The plant materials were collected from Duntou Village (117°51′, 27°23′), Mashan Town, Jianyang District, Nanping City, Fujian Province, China, and authenticated by Senior Agronomist Yao Changchun from the Rural Revitalization Development Center of Mashan Town, Jianyang District, Nanping City. A voucher specimen (#20220422) was deposited in the Fujian Key Laboratory of Medical Analysis, Fujian Academy of Medical Sciences. Powdered samples (40-mesh) of *A. japonica* fruits (SJGS), rhizomes (SJGJ), and leaves (SJY) (30 g each) underwent sequential ethanol extraction (60%) by steam distillation reflux: primary extraction with 600 mL (90 min) followed by secondary extraction with 300 mL (60 min). After filtration, extracts were concentrated to 0.5 g/mL (*w*/*v*, crude drug equivalent).

All commercial kits and reagents were obtained from reputable suppliers. The biochemical assay kits for Total Cholesterol (TC, A111-1-1), Triglycerides (TG, A110-1-1), High-Density Lipoprotein Cholesterol (HDL, A112-1-1), Low-Density Lipoprotein Cholesterol (LDL, A113-1-1), Alanine Aminotransferase (ALT, C009-2-1), and Aspartate Aminotransferase (AST, C010-2-1) were obtained from Nanjing Jiancheng Bioengineering Institute (Nanjing, China), while the Rat Diamine Oxidase (DAO) ELISA Kit (BYHS101460) was acquired from Nanjing Boyan Biotechnology (Nanjing, China). RNA extraction was performed using the RC112-01 kit (Vazyme Biotech, Nanjing, China), followed by reverse transcription with the AT341-02 kit (Beijing TransGen Biotech, Beijing, China) and quantitative PCR using the Q712 kit (Nanjing Vazyme Biotech, Nanjing, China). Histological procedures employed xylene (10023418), *n*-butanol (100052190), and neutral balsam (10004160) from Sinopharm Chemical Reagent Co., Ltd. (Shanghai, China), along with eco-friendly dewaxing solution (G1128), universal tissue fixative (G1101), and HE staining kit (G1076) from Servicebio (Wuhan, China). Additional reagents included ethanol (Xilong Scientific Co., Ltd., Guangzhou, China), DEPC water (R0022, Beyotime Biotechnology, Shanghai, China), and the HiPure Stool DNA Kit (D3141-02B, Magen BioSciences, Guangzhou, China) for microbial DNA extraction.

### 4.2. Experimental Design and Animal Handling

Forty male Sprague Dawley rats (6–8 weeks old, 180–220 g) were housed under controlled conditions (23 ± 3 °C, 50–60% humidity, 12 h light/12 h dark) with free access to water. After 7-day acclimation, rats were assigned to two groups: the control group was fed regular chow (containing soybean oil, corn, fish meal, etc.) and model group fed high-fat/high-sucrose/high-salt diet (60.5% base feed supplemented with 10% lard, 12% sucrose, 12% egg yolk, 0.5% sodium cholate, 2% NaCl and 3% cholesterol). All procedures were performed in accordance with institutional animal care guidelines.

Following a 1-week acclimation period, Sprague Dawley rats were randomly assigned to a normal control group (CON, n = 8) and model (n = 32) groups. The CON group was allotted standard chow, while the model group was fed a high-fat/high-sucrose/high-salt diet and housed in humidity-controlled cages to establish a “dietary irregularity” model mimicking traditional Chinese medicine pathogenesis. Over 8 weeks, model rats underwent alternating interventions: lard gavage (2 mL on odd days) and cold water stress (4 °C, 10 mL/kg on even days), with daily monitoring of physiological parameters. After 4 weeks of modeling, successfully induced rats were weight-stratified into model control (MOD), *A. japonica* fruit- (SJGS), rhizome- (SJGJ), and leaf-treated (SJY) groups (n = 8 each). From weeks 5–8, treatment groups received medium-dose extracts (0.9 g/kg, calculated as 6.3× human equivalent dose) via daily afternoon gavage, while CON and MOD groups received equal-volume saline, with weekly body weight measurements for dose adjustment.

### 4.3. Sample Collection and Parameter Analysis

Following 4 weeks of treatment, biological samples were systematically collected, including fecal specimens (flash-frozen for microbiome analysis), fasting serum (obtained via abdominal aortic puncture after 12 h fasting), and tissue samples (liver and epididymal fat pads for histopathological and molecular analyses). Intestinal motility was assessed by carbon transit measurement prior to sacrifice, with ileal segments preserved for gene expression studies. The comprehensive physiological monitoring protocol encompassed daily assessments of general health status, weekly anthropometric measurements (including body weight, length, and abdominal circumference), triplicate blood pressure measurements obtained through tail-cuff plethysmography, and biweekly fasting glucose determinations. Biochemical analyses encompassed serum lipid levels (TC, TG, HDL-C, and LDL-C), hepatic health markers (AST, ALT), and intestinal permeability indicator (DAO) using standardized assay kits. Histopathological examination of liver tissues employed conventional H&E staining.

### 4.4. Analysis of Gut Microbiota Abundance and Diversity

Fresh fecal samples from rats were collected under sterile conditions using cryotubes, rapidly frozen in liquid nitrogen, and stored at −80 °C pending analysis. Following collection, samples were transported to Biomarker Technologies Co., Ltd. (Beijing, China) for Illumina-based sequencing of bacterial 16S rRNA gene V3-V4 regions. Total genomic DNA was isolated using the HiPure Stool DNA Kit, followed by quality assessment through spectrophotometry and agarose gel electrophoresis to verify concentration, purity, and integrity. The diluted DNA was then used as a template for PCR amplification of the target 16S rDNA region with specific primers. The resulting PCR products were purified, quantified, and pooled in equimolar ratios for library construction with sequencing adapters. Finally, paired-end sequencing was performed on the Illumina platform.

For bioinformatics analysis, raw sequencing data were processed using QIIME2 and the DADA2 plugin to filter low-quality reads and generate amplicon sequence variants (ASVs). Alpha diversity (within-community diversity) and beta diversity (between-community differences) indices were calculated using R and QIIME2. Taxonomic classification was performed at the phylum, family, and genus levels, and microbial community composition was visualized using stacked bar plots to highlight structural variations across different taxonomic ranks.

### 4.5. Ileal Transcriptome Sequencing and Analysis

The experimental procedure involved ileal transcriptome profiling, beginning with RNA extraction and quality assessment to ensure suitability for downstream applications. Eukaryotic mRNA was captured by poly(dT) magnetic bead selection, followed by fragmentation and cDNA synthesis through reverse transcription. Following end-repair and A-tailing, adapters were ligated to the cDNA, which was then size-selected and PCR-amplified to generate sequencing libraries. Following end-repair and A-tailing, adapters were ligated to the cDNA. The resulting libraries were size-selected, PCR-amplified, and then rigorously quantified (Qubit 3.0) and size-verified (Qsep400) to meet sequencing quality standards. Qualified libraries were processed on an Illumina HiSeq 4000 platform, and the primary data were analyzed through the BMKCloud pipeline for quality control, reference genome alignment, and transcript quantification using StringTie to determine gene expression levels in RPKM values. Differential gene expression analysis was performed with a threshold of |FC| ≥ 1.5 and FDR < 0.05, with results visualized in volcano plots, followed by comprehensive functional annotation through GO enrichment and pathway analysis using KEGG to elucidate the biological significance of the identified differentially expressed genes (DEGs).

### 4.6. Quantitative Real-Time PCR Analysis of Ileal mRNA Expression

Quantitative real-time PCR (qRT-PCR) was performed to determine the mRNA expression levels of target genes in ileal tissues. Total RNA was reverse transcribed into cDNA using TransScript^®^ First-Strand cDNA Synthesis SuperMix (TransGen Biotech, Beijing, China) according to the manufacturer’s protocol, with the following thermal cycling conditions: 42 °C for 15 min, 85 °C for 5 s, followed by cooling to 20 °C. The 20 μL reaction mixture contained 4 μL 5×TransScript^®^ All-in-One SuperMix, 1 μg total RNA, and nuclease-free water. GAPDH was selected as the endogenous reference gene for normalization. Target gene-specific primers (Table 2) were designed and synthesized commercially. qPCR amplification was carried out in a 20 μL reaction volume containing 10 μL qPCR Master Mix (Vazyme, China). The amplification process comprised an initial denaturation phase at 95 °C for 30 s, followed by 40 amplification cycles, each consisting of 10 s at 95 °C and 30 s at 60 °C. Melting curve analysis was performed to validate the specificity of the amplification reaction. The 2^−ΔΔCt^ method was employed to calculate relative gene expression levels.

### 4.7. Chemical Profiling by UPLC-Q-Exactive-Orbitrap-MS

The chemical constituents of *A. japonica* fruits, rhizomes, and leaves were analyzed using ultra-performance liquid chromatography coupled with quadrupole-Orbitrap mass spectrometry (UPLC-Q-Exactive-Orbitrap-MS, Thermo Scientific, Waltham, MA, USA). Powdered samples (0.5 g, 40 mesh) were extracted twice with 60% ethanol (10 mL) under reflux (first extraction: 1 h; second extraction: 30 min). The combined filtrates were adjusted to 25 mL and filtered (0.22 μm) prior to analysis. Chromatographic separation was performed using an ACQUITY UPLC^®^ BEH C18 column (100 × 2.1 mm, 1.7 μm; Waters, Milford, MA, USA) maintained at 40 °C. The mobile phase comprised (A) 0.1% formic acid in water and (B) pure acetonitrile (not acid-modified), delivered at 0.2 mL/min with the following gradient: 5% B (0–0.1 min), 5–30% B (0.1–4 min), 30–60% B (4–8 min), 60–90% B (8–11 min), isocratic 90% B (11–12.5 min), returning to 5% B (12.5–14.5 min), and re-equilibration (14.5–17 min). Mass detection employed a heated electrospray ionization (HESI) source operating in dual-polarity mode with the following parameters: spray voltage 3.2 kV; sheath/auxiliary gas flows 45/10 (arbitrary units); capillary temperature 325 °C; auxiliary gas heater 350 °C. Full-scan data (100–1200 m/z) were acquired at 70,000 resolution (AGC target 3e^6^, maximum IT 100 ms). Chemical constituents identification was conducted by comparing exact mass (mass error < 5 ppm) and MS/MS fragmentation patterns against the Thermo Scientific mzVault Library. Identifications were assigned confidence levels following the Metabolomics Standards Initiative [64].

### 4.8. Network Pharmacology Analysis

The network pharmacology approach was implemented to investigate the potential anti-hyperlipidemic mechanisms of *A. japonica*. Active components from different plant parts were identified by screening their Isomeric SMILES structures (obtained from PubChem) using the SwissADME web tool (http://www.swissadme.ch, accessed on 17 December 2024), with selection criteria including high gastrointestinal absorption and compliance with ≥2 Lipinski’s rule of five parameters. Potential targets were predicted via SwissTargetPrediction (human species, probability threshold >0.1). Hyperlipidemia-related targets were retrieved from GeneCards (Relevance score ≥1) and intersected with the component targets using a Venn diagram (Weishengxin platform, https://www.bioinformatics.com.cn/, accessed on 18 December 2024) to identify potential therapeutic targets. A compound-target network was constructed using Cytoscape 3.9.1, with core active components selected based on degree values. Protein–protein interaction (PPI) networks were constructed using the STRING database (human, confidence score >0.4), followed by core target screening through degree analysis. Functional enrichment analysis was conducted employing Metascape for both GO terms and KEGG pathways (*p* < 0.01), with results visualized through the Weishengxin platform.

### 4.9. Statistical Analysis

All experimental data were processed with GraphPad Prism 8 (GraphPad Software, San Diego, CA, USA). Quantitative variables are presented as mean ± standard deviation (SD). The normality of data distribution was assessed using the Shapiro–Wilk test. Homogeneity of variances was verified by Levene’s test. Multiple-group comparisons were performed using one-way analysis of variance (ANOVA), with subsequent pairwise comparisons conducted via the LSD post hoc test. A two-tailed *p*-value less than 0.05 was considered to indicate statistical significance. Compound–gene expression correlations were evaluated using Pearson’s coefficient through the OmicShare Tools platform [65] (Guangzhou Gidio Biotechnology Co., Ltd., Guangzhou, China). Statistical significance was defined as *p* < 0.05, with |r| ≥ 0.7 considered biologically relevant.

## 5. Conclusions

This study systematically evaluates the hypolipidemic effects of *A. japonica* fruit, rhizome, and leaf extracts, demonstrating their shared efficacy but distinct mechanisms. Fruit and rhizome extracts appear to act through hepatic fatty acid oxidation genes (*ACSL1*, *ACOX1*), while leaves may primarily affect intestinal APOA1 production. Integrated LC-MS and network pharmacology analyses pinpointed pinocembrin and galangin as potential key flavonoids that could contribute to PPAR pathway activation.

## Figures and Tables

**Table 1:** DEGs regulation by *A. japonica* in high-fat diet-fed rats’ Ileum.

Groups	Number of DEGs		DEGs in Lipid Metabolic Pathways
CON vs. MOD	1310	↑719	*SCD2, FAdS2, SCD1, ABCG1, ABCA2, SOCS3, LEPR, PI3K, IL-6R*
		↓591	*FABP1, PCK1, ACADM, PPARG, FABP2, ACOX1, ACSL1, ACADL PLIN1, ADIPOQ, APOC2*
SJGJ vs. MOD	976	↑237	*——*
		↓739	*FABP1, APOA1, AQP7, PCK1, APOC3, ACADM, ACOX1, FABP2, ACSL1, PLIN1, ADIPOQ, APOC2, GK, PCK1, ACOX3*
SJY vs. MOD	319	↑176	*——*
		↓143	*APOA1, APOC3, CREB3l3, APOC2*
SJGS vs. MOD	3587	↑1692	*LPL HMGCS2, SCD, ABCG1, SCD2, SCD1, PLTP, AKT3, LEPR, IL-6R, NFKBb1, CPT1C*
		↓1895	*ACOX1, APOC3, APOA1, PCK1, FABP1, ACSL1, PPARA, AQP7, FABP2, ACADM, SREBF1, PFKFB4, G6PC, PCK1, HNF4A, FBP1, LIPE, RXRA*

Note: ↑ indicates up-regulated genes, ↓ indicates down-regulated genes, and — represents non-DEGs in lipid metabolic pathways.

**Table 2:** Primer sequences used for qRT-PCR analysis.

No.	Gene Symbol	Primer Sequence (5′→3′)	Length (bp)
1	*GAPDH*-F	ACGGCAAGTTCAACGGCACAG	21
2	*GAPDH*-R	GAAGACGCCAGTAGACTCCACGAC	24
3	*ZO-1*-F	AGTCCCTTACCTTTCGCCTG	20
4	*ZO-1*-R	CAGCTTGTGATACGTGCGAG	20
5	*OCCLUDIN*-F	CCACCTATCACTTCAGATCAACAG	24
6	*OCCLUDIN*-R	ATGTACTCTTCGCTCTCCTCTC	22
7	*CLAUDIN*-F	GGTTGCAGCTTCTGGGTTTC	20
8	*CLAUDIN*-R	CAGTCACGATGTTGTCCCCA	20
9	*APOAL*-F	GAGGAGTTTCGAGACCGCAT	20
10	*APOAL*-R	GTCGCTGGCCTTGGTATGAT	20
11	*ACADM*-F	TCACCGGATTCATCGTGGAG	20
12	*ACADM*-R	CGTCGGCCTGGTTCTATCAA	20
13	*ACOX1*-F	TCACTCGAAGCCAGCGTTAT	20
14	*ACOX1*-R	AGCAGTGGTGCCCTGATTC	19
15	*ACSL1*-F	CAAGGTGCTTCAGCCTACCA	20
16	*ACSL1*-R	ATCCAACAGCCATCGCTTCA	20

## Data Availability

The raw LC-MS data and transcriptome sequencing reads generated in this study have been deposited in public repositories. LC-MS metabolomics data: [MetaboLights] under accession number [12910]; RNA-seq data: [NCBI SRA] under BioProject ID [PRJNA1304238]. Processed data supporting the findings are included in the article and its Supplementary Materials.

## Supplementary Materials

The following supporting information can be downloaded at: https://www.mdpi.com/article/10.3390/ph18091320/s1, Table S1: Statistics of sample sequencing data processing results; Table S2: Distribution table of feature counts for each sample; Table S3: Identification of Chemical Constituents in *A. japonica* by UPLC-Q-Exactive Orbitrap-MS.

## References

[1] Editorial Committee of Flora of China (1981). Flora Reipublicae Popularis Sinicae.

[2] Qiu S. (1996). Textual Research on Medicinal Alpinia (Shanjiang). J. Fujian Coll. Tradit. Chin. Med..

[3] Zhao J., Xu T., Luo J., Peng Q., Gao Z., Xiang J. (2024). Effects of Volatile Oil of the *Alpinia japonica* Rhizome on Gastrointestinal Motility of Mice in Tujia Medicine. J. Jishou Univ. (Nat. Sci. Ed.).

[4] Asakawa Y., Ludwiczuk A., Sakurai K., Tomiyama K., Kawakami Y., Yaguchi Y. (2017). Comparative Study on Volatile Compounds of *Alpinia japonica* and *Elettaria cardamomum*. J. Oleo Sci..

[5] Kim J.-G., Kim W., Kim K.-Y. (2023). *Alpinia japonica* Extract Induces Apoptosis of Hepatocellular Carcinoma Cells through G0/G1 Cell Cycle Arrest and Activation of JNK. Cell. Mol. Biol..

[6] Li Q.-M., Luo J.-G., Yang M.-H., Kong L.-Y. (2015). Terpenoids from Rhizomes of *Alpinia japonica* Inhibiting Nitric Oxide Production. Chem. Biodivers..

[7] Li Q.-M., Luo J.-G., Wang X.-B., Yang M.-H., Kong L.-Y. (2013). Sesquiterpenes from the Rhizomes of *Alpinia japonica* and Their Inhibitory Effects on Nitric Oxide Production. Fitoterapia.

[8] Kakegawa T., Miyazaki A., Yasukawa K. (2016). Anti-Inflammatory Effects of Alpinone 3-Acetate from *Alpinia japonica* Seeds. J. Nat. Med..

[9] Mei L.-N., Wang Z.-J., Duan Y., Shen J.-S., Ye H.-B., Zhu Y.-Y., Luo X.-D. (2025). 4-Hydroxyboesenbergin B of *Alpinia japonica* Protected Gastrointestinal Tract by Inhibiting Vancomycin-Resistant Enterococcus and Balancing Intestinal Microbiota. J. Ethnopharmacol..

[10] You M., Zhou L., Wu F., Zhang L., Zhu S.-X., Zhang H.-X. (2025). Probiotics for the Treatment of Hyperlipidemia: Focus on Gut-Liver Axis and Lipid Metabolism. Pharmacol. Res..

[11] Abbasi S., Khan A., Choudhry M.W. (2024). New Insights Into the Treatment of Hyperlipidemia: Pharmacological Updates and Emerging Treatments. Cureus.

[12] Cholesterol Treatment Trialists’ (CTT) Collaboration (2024). Effects of Statin Therapy on Diagnoses of New-Onset Diabetes and Worsening Glycaemia in Large-Scale Randomised Blinded Statin Trials: An Individual Participant Data Meta-Analysis. Lancet Diabetes Endocrinol..

[13] He K., Hu Y., Ma H., Zou Z., Xiao Y., Yang Y., Feng M., Li X., Ye X. (2016). Rhizoma Coptidis Alkaloids Alleviate Hyperlipidemia in B6 Mice by Modulating Gut Microbiota and Bile Acid Pathways. Biochim. Biophys. Acta (BBA) Mol. Basis Dis..

[14] Qiu J., Chen L., Zhang L., Xu F., Zhang C., Ren G., Chang K., He G., Du Z., Le Y. (2023). Xie Zhuo Tiao Zhi Formula Modulates Intestinal Microbiota and Liver Purine Metabolism to Suppress Hepatic Steatosis and Pyroptosis in NAFLD Therapy. Phytomedicine.

[15] Heidari H., Khalaj A., Khani S., Abdollahi M., Farahani H., Khani S. (2022). Hypoglycemic, Hypolipidemic and Hepatoprotective Effects of *Alpinia officinarum* on Nicotinamide/Streptozotocin Induced Type II Diabetic Rats. Horm. Mol. Biol. Clin. Investig..

[16] Xia D.-Z., Yu X.-F., Wang H.-M., Ren Q.-Y., Chen B.-M. (2010). Anti-Obesity and Hypolipidemic Effects of Ethanolic Extract from *Alpinia officinarum* Hance (Zingiberaceae) in Rats Fed High-Fat Diet. J. Med. Food.

[17] Xiao T., Huang J., Wang X., Wu L., Zhou X., Jiang F., He Z., Guo Q., Tao L., Shen X. (2020). *Alpinia zerumbet* and Its Potential Use as an Herbal Medication for Atherosclerosis: Mechanistic Insights from Cell and Rodent Studies. Lifestyle Genom..

[18] Chuang C.-M., Wang H.-E., Peng C.-C., Chen K.C., Peng R.Y. (2011). Hypolipidemic Effects of Different Angiocarp Parts of *Alpinia zerumbet*. Pharm. Biol..

[19] Rampanelli E., Romp N., Troise A.D., Ananthasabesan J., Wu H., Gül I.S., De Pascale S., Scaloni A., Bäckhed F., Fogliano V. (2025). Gut Bacterium *Intestinimonas butyriciproducens* Improves Host Metabolic Health: Evidence from Cohort and Animal Intervention Studies. Microbiome.

[20] Schoeler M., Ellero-Simatos S., Birkner T., Mayneris-Perxachs J., Olsson L., Brolin H., Loeber U., Kraft J.D., Polizzi A., Martí-Navas M. (2023). The Interplay between Dietary Fatty Acids and Gut Microbiota Influences Host Metabolism and Hepatic Steatosis. Nat. Commun..

[21] Won T.H., Arifuzzaman M., Parkhurst C.N., Miranda I.C., Zhang B., Hu E., Kashyap S., Letourneau J., Jin W.-B., Fu Y. (2025). Host Metabolism Balances Microbial Regulation of Bile Acid Signalling. Nature.

[22] Du H., Shi L., Wang Q., Yan T., Wang Y., Zhang X., Yang C., Zhao Y., Yang X. (2022). Fu Brick Tea Polysaccharides Prevent Obesity via Gut Microbiota-Controlled Promotion of Adipocyte Browning and Thermogenesis. J. Agric. Food Chem..

[23] Manzo R., Gallardo-Becerra L., Díaz de León-Guerrero S., Villaseñor T., Cornejo-Granados F., Salazar-León J., Ochoa-Leyva A., Pedraza-Alva G., Pérez-Martínez L. (2024). Environmental Enrichment Prevents Gut Dysbiosis Progression and Enhances Glucose Metabolism in High-Fat Diet-Induced Obese Mice. Int. J. Mol. Sci..

[24] Yan S., Zhou J., Zhang H., Lin Z., Khambu B., Liu G., Ma M., Chen X., Chalasani N., Yin X.-M. (2022). Promotion of Diet-Induced Obesity and Metabolic Syndromes by BID Is Associated with Gut Microbiota. Hepatol. Commun..

[25] Cai R., Yue X., Wang Y., Yang Y., Sun D., Li H., Chen L. (2021). Chemistry and Bioactivity of Plants from the Genus. Amomum. J. Ethnopharmacol..

[26] Cai W., Jiang L., Zhao C., Zhou X. (2025). Advances in Omics Technologies for Traditional Chinese Medicine in the Prevention and Treatment of Metabolic Bone Diseases. Front. Pharmacol..

[27] Fan Y., Pedersen O. (2021). Gut Microbiota in Human Metabolic Health and Disease. Nat. Rev. Microbiol..

[28] Wang B., Zhang B., Zhou L., Li S., Li Z., Luo H. (2023). Multi-Omics Reveals Diet-Induced Metabolic Disorders and Liver Inflammation via Microbiota-Gut-Liver Axis. J. Nutr. Biochem..

[29] Peng L., Li H., Yang L., Liang Z., Zhang X. (2025). Exploring the Metabolic and Transcriptomic Profiles of *Tetrastigma hemsleyanum* for Tissue-Specific Compound Accumulation. Front. Plant Sci..

[30] Maslahat M., Mardinata D., Surur S.M., Lioe H.N., Syafitri U.D., Rafi M., Rohaeti E. (2025). Untargeted Metabolomics Analysis Using FTIR and LC-HRMS for Differentiating Sonchus Arvensis Plant Parts and Evaluating Their Biological Activity. Chem. Biodivers..

[31] Yang W., Liu F., Wu G., Liang S., Bai X., Liu B., Zhang B., Chen H., Yang J. (2024). Widely Targeted Metabolomics Analysis of the Roots, Stems, Leaves, Flowers, and Fruits of *Camellia luteoflora*, a Species with an Extremely Small Population. Molecules.

[32] Farag M.A., Baky M.H., Morgan I., Khalifa M.R., Rennert R., Mohamed O.G., El-Sayed M.M., Porzel A., Wessjohann L.A., Ramadan N.S. (2023). Comparison of Balanites Aegyptiaca Parts: Metabolome Providing Insights into Plant Health Benefits and Valorization Purposes as Analyzed Using Multiplex GC-MS, LC-MS, NMR-Based Metabolomics, and Molecular Networking. RSC Adv..

[33] Amari N.O., Missoun F., Chaimbault P., Berkani A. (2022). Profiling by LC-MS and LC-MS/MS with Electrospray source (ESI) of extracts from three organs of *Thymelaea hirsuta* L.. Ann. Pharm. Fr..

[34] Curtis P.J., Berends L., van der Velpen V., Jennings A., Haag L., Chandra P., Kay C.D., Rimm E.B., Cassidy A. (2022). Blueberry Anthocyanin Intake Attenuates the Postprandial Cardiometabolic Effect of an Energy-Dense Food Challenge: Results from a Double Blind, Randomized Controlled Trial in Metabolic Syndrome Participants. Clin. Nutr..

[35] Xu Y., Wang M., Luo Y., Liu H., Ling H., He Y., Lu Y. (2024). PPARα Is One of the Key Targets for Dendrobine to Improve Hepatic Steatosis in NAFLD. J. Ethnopharmacol..

[36] Jung J.-W., Wang F., Turk A., Park J.-S., Ma H., Ma Y., Noh H.-R., Sui G., Shin D.-S., Lee M.-K. (2023). Zaluzanin C Alleviates Inflammation and Lipid Accumulation in Kupffer Cells and Hepatocytes by Regulating Mitochondrial ROS. Molecules.

[37] Zhou T., Yan K., Zhang Y., Zhu L., Liao Y., Zheng X., Chen Y., Li X., Liu Z., Zhang Z. (2022). Fenofibrate Suppresses Corneal Neovascularization by Regulating Lipid Metabolism through PPARα Signaling Pathway. Front. Pharmacol..

[38] Gerges S.H., Wahdan S.A., Elsherbiny D.A., El-Demerdash E. (2021). Non-Alcoholic Fatty Liver Disease: An Overview of Risk Factors, Pathophysiological Mechanisms, Diagnostic Procedures, and Therapeutic Interventions. Life Sci..

[39] Kuang J., Wang J., Li Y., Li M., Zhao M., Ge K., Zheng D., Cheung K.C.P., Liao B., Wang S. (2023). Hyodeoxycholic Acid Alleviates Non-Alcoholic Fatty Liver Disease through Modulating the Gut-Liver Axis. Cell Metab..

[40] Sharma S., Sharma A., Chauhan R.S. (2024). Computational Dissection through Network Pharmacology and Structure-Based Analysis Unravels Mechanistic Actions of Bioactive Compounds in a Hepatoprotective Herb, *Picrorhiza kurroa* for the Treatment of NAFLD and NASH. J. Biomol. Struct. Dyn..

[41] Boniface P.K., Fabrice F.B., Paumo H.K., Katata-Seru L.M. (2023). Protective Roles and Mechanism of Action of Plant Flavonoids against Hepatic Impairment: Recent Developments. Curr. Drug Targets.

[42] Jinadatta P., Rajshekarappa S., Sundera Raja Rao K., Pasura Subbaiah S.G., Shastri S. (2019). In Silico, in Vitro: Antioxidant and Antihepatotoxic Activity of Gnetol from *Gnetum ula* Brongn. BioImpacts.

[43] Li S., Duan F., Li S., Lu B. (2024). Administration of Silymarin in NAFLD/NASH: A Systematic Review and Meta-Analysis. Ann. Hepatol..

[44] Calle R.A., Amin N.B., Carvajal-Gonzalez S., Ross T.T., Bergman A., Aggarwal S., Crowley C., Rinaldi A., Mancuso J., Aggarwal N. (2021). ACC Inhibitor Alone or Co-Administered with a DGAT2 Inhibitor in Patients with Non-Alcoholic Fatty Liver Disease: Two Parallel, Placebo-Controlled, Randomized Phase 2a Trials. Nat. Med..

[45] Hadjihambi A., Konstantinou C., Klohs J., Monsorno K., Le Guennec A., Donnelly C., Cox I.J., Kusumbe A., Hosford P.S., Soffientini U. (2023). Partial MCT1 Invalidation Protects against Diet-Induced Non-Alcoholic Fatty Liver Disease and the Associated Brain Dysfunction. J. Hepatol..

[46] Kennedy L., Meadows V., Sybenga A., Demieville J., Chen L., Hargrove L., Ekser B., Dar W., Ceci L., Kundu D. (2021). Mast Cells Promote Nonalcoholic Fatty Liver Disease Phenotypes and Microvesicular Steatosis in Mice Fed a Western Diet. Hepatology.

[47] Liu Q., Xu Y., Lv X., Guo C., Zhu H., Yang L., Wang Y. (2024). 2′, 3′, 5′-Tri-*O*-acetyl-*N*6-(3-hydroxyphenyl) Adenosine Alleviates Diet-Induced Hyperlipidemia by Modulating Intestinal Gene Expression Profiles and Metabolic Pathway. Life Sci..

[48] Manaer T., Sailike J., Sun X., Yeerjiang B., Nabi X. (2024). Therapeutic Effects of Composite Probiotics Derived from Fermented Camel Milk on Metabolic Dysregulation and Intestinal Barrier Integrity in Type 2 Diabetes Rats. Front. Pharmacol..

[49] Chen Y., Shen P., Li Q., Ong S.S., Qian Y., Lu H., Li M., Xu T. (2025). Electroacupuncture and Tongbian Decoction Ameliorate CUMS-Induced Depression and Constipation in Mice via TPH2/5-HT Pathway of the Gut-Brain Axis. Brain Res. Bull..

[50] Liu X., Luo M., Wang Z., Yang S.J., Su M., Wang Y., Wang W., Sun Z., Cai Y., Wu L. (2024). Mind Shift I: Fructus Aurantii–Rhizoma Chuanxiong Synergistically Anchors Stress-Induced Depression-like Behaviours and Gastrointestinal Dysmotility Cluster by Regulating Psycho-Immune-Neuroendocrine Network. Phytomedicine.

[51] Zhang Y.-X., Zhang Y.-J., Li M., Tian J.-X., Tong X.-L. (2024). Common Pathophysiological Mechanisms and Treatment of Diabetic Gastroparesis. J. Neurogastroenterol. Motil..

[52] Li X., Liu Y., Liu N., Wu H., Cong K., Duan L., Chen T., Zhang J. (2025). Health Benefits of Medicinal Plant Natural Products via Microbiota-Mediated Different Gut Axes. Pharmacol. Res..

[53] Xie P., Ma Z., Yang X., Pan H., Shen P., Du H., Cao Y., Xiao J., Xiao H., Song M. (2025). The Regulatory Effects and Mechanisms of Plant Food-Derived Bioactive Components on Gut Barrier Function and Intestinal Homeostasis: A Comprehensive Review. Crit. Rev. Food Sci. Nutr..

[54] Cullen J.M.A., Shahzad S., Dhillon J. (2023). A Systematic Review on the Effects of Exercise on Gut Microbial Diversity, Taxonomic Composition, and Microbial Metabolites: Identifying Research Gaps and Future Directions. Front. Physiol..

[55] García-Gamboa R., Díaz-Torres O., Senés-Guerrero C., Gradilla-Hernández M.S., Moya A., Pérez-Brocal V., Garcia-Gonzalez A., González-Avila M. (2024). Associations between Bacterial and Fungal Communities in the Human Gut Microbiota and Their Implications for Nutritional Status and Body Weight. Sci. Rep..

[56] Al-Adham I.S.I., Agha A.S.A.A., Al-Akayleh F., Al-Remawi M., Jaber N., Al Manasur M., Collier P.J. (2025). Prebiotics Beyond the Gut: Omics Insights, Artificial Intelligence, and Clinical Trials in Organ-Specific Applications. Probiotics Antimicrob. Proteins.

[57] Ren L., Ma X.-L., Wang H.-L., Li R., Cui J.-J., Yan P.-J., Wang Y.-N., Yu X.-Y., Du P., Yu H.-Y. (2022). Prebiotic-like Cyclodextrin Assisted Silybin on NAFLD through Restoring Liver and Gut Homeostasis. J. Control. Release.

[58] Cao P., Chen Q., Shi C., Pei M., Wang L., Gong Z. (2022). Pinocembrin Ameliorates Acute Liver Failure via Activating the Sirt1/PPARα Pathway in Vitro and in Vivo. Eur. J. Pharmacol..

[59] Kumar S., Alagawadi K.R. (2013). Anti-Obesity Effects of Galangin, a Pancreatic Lipase Inhibitor in Cafeteria Diet Fed Female Rats. Pharm. Biol..

[60] Zhou Y., Ding Y.-L., Zhang J.-L., Zhang P., Wang J.-Q., Li Z.-H. (2018). Alpinetin Improved High Fat Diet-Induced Non-Alcoholic Fatty Liver Disease (NAFLD) through Improving Oxidative Stress, Inflammatory Response and Lipid Metabolism. Biomed. Pharmacother..

[61] Wang T., He C. (2018). Pro-Inflammatory Cytokines: The Link between Obesity and Osteoarthritis. Cytokine Growth Factor. Rev..

[62] Bu L., Zhang Z., Chen J., Fan Y., Guo J., Su Y., Wang H., Zhang X., Wu X., Jiang Q. (2024). High-Fat Diet Promotes Liver Tumorigenesis via Palmitoylation and Activation of AKT. Gut.

[63] Wang S.-Q., Xiang J., Zhang G.-Q., Fu L.-Y., Xu Y.-N., Chen Y., Tao L., Hu X.-X., Shen X.-C. (2024). Essential Oil from Fructus *Alpinia zerumbet* Ameliorates Atherosclerosis by Activating PPARγ-LXRα-ABCA1/G1 Signaling Pathway. Phytomedicine.

[64] Schymanski E.L., Jeon J., Gulde R., Fenner K., Ruff M., Singer H.P., Hollender J. (2014). Identifying Small Molecules via High Resolution Mass Spectrometry: Communicating Confidence. Environ. Sci. Technol..

[65] Mu H., Chen J., Huang W., Huang G., Deng M., Hong S., Ai P., Gao C., Zhou H. (2024). OmicShare Tools: A Zero-code Interactive Online Platform for Biological Data Analysis and Visualization. iMeta.

